# Unusual Postrhinoplasty Complication: Nasal Dorsum Cyst

**DOI:** 10.1155/2014/617424

**Published:** 2014-09-09

**Authors:** Pier Giorgio Giacomini, Davide Topazio, Roberta Di Mauro, Stelio Mocella, Matteo Chimenti, Stefano Di Girolamo

**Affiliations:** ^1^Department of Otorhinolaryngology, University of Rome “Tor Vergata”, Rome, Italy; ^2^Department of Otorhinolaryngology, Bussolengo Hospital, Italy; ^3^Department of Pathology, University of Rome “Tor Vergata”, Rome, Italy

## Abstract

Among all the possible complications of aesthetic rhinoplasty, a rare one is the development of cystic masses on the nasal dorsum: several theories suggest that cysts develop commonly by entrapment of nasal mucosa in the subcutaneous space, but they can also originate from foreign body reactions. This report deals with two cases of nasal dorsum cysts with different pathogenesis: both patients had undergone aesthetic rhinoplasty in the past (26 years ago and 14 years ago, resp.). Both cystic masses were removed via a direct open approach and nasal reconstruction was performed successfully with autologous vomer bone. The pathologic investigations showed a foreign body inclusion cyst associated with latex rubber in the first case and a sequestration of a mucosal-lined nasal bone was not removed at the time of primary rhinoplasty in the second case. A brief review of the literature focuses on the pathophysiology and treatment options for nasal dorsal cysts following aesthetic rhinoplasty.

## 1. Introduction

The development of postrhinoplasty nasal dorsal irregularities due to bone or cartilaginous bossae is a common complication, whereas cysts formation is a rare event. Reviewing previously reported cases reveals that mucous cysts are by far most prevalent in the nasal dorsum. Other locations include the nasal tip, the medial canthus, and the paranasal area [1–3]. Probably, migration or incorporation of mucosal tissue (or bony or cartilaginous fragments not accurately removed during primary surgery in the subcutaneous space) commonly accounts for cyst formation [4].

With regard to nonmucosal cysts of the subcutaneous area, a fully different pathophysiology, namely, foreign body reaction, may be considered. Petrolatum jelly migrated from the packing material and foreign body reaction to alloimplants employed for augmentation have been reported to be possible causes [5]. Other rare pathogenetic mechanisms like congenital malformations in cleft lip nose rhinoplasty or remnants of the nasolacrimal duct have been described [6, 7].

Since the postsurgical cyst development could be in part prevented, particularly by considering all pathogenetic mechanisms, this report aims at underlining the need of further insight into this rare entity.

## 2. Case 1

We evaluated a 62-year-old Caucasian male with an asymptomatic nasal radix mass and nasal airflow obstruction. Twenty-six years ago he had undergone reduction rhinoplasty with an uneventful postoperative period; however, nasal obstruction had not improved.

**Figure 1:**
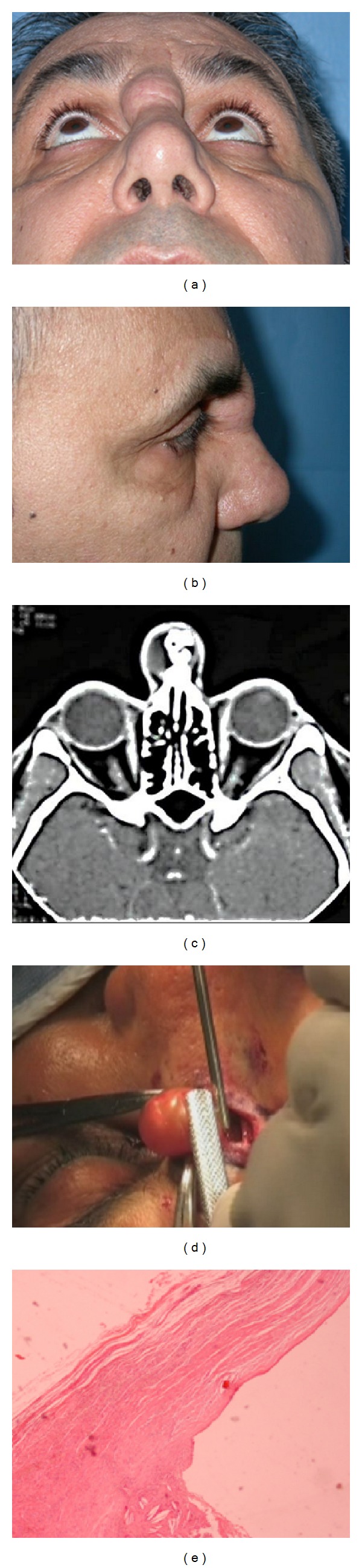
(a) Frontal view (Patient #1), (b) lateral view (Patient #1), (c) preoperative CT scan (Patient #1), (d) intraoperative view of nasal cyst removal (Patient #1), and (e) pathology specimen (Patient #1).

A nasal dorsum mass, measuring 5 × 5 mm was noted three months postoperatively and slowly increased in size over the years. On physical examination, the nasal dorsum and tip were firm on palpation and a small deformity was present in the upper nasal dorsum, appearing as a 2.0 × 2.0 cm round, soft, and painless mass located on the midline of nasal radix (Figure 1(a)). A computerized tomography (CT) scan was conducted to exclude possible bone involvement and revealed a heterogeneous, large cystic mass on the nasal radix, measuring 68 Hounsfield units (HU), adhering without erosion to the nasal bones cranial part. It contained a cystic aspect including a homogenous density foreign body (Figure 1(b)), which was first thought to be a silicone implant.

In consideration of clinical and CT-features, a presurgical diagnosis of nasal radix cyst as a consequence of previous augmentation rhinoplasty was made. The mass was successfully removed via a horizontal glabellar incision. After a skin incision, the cyst was dissected down to the nasal bone and removed in en-bloc fashion because of its close relation to the bony dorsum (Figure 1(c)).

The removed cyst contained a liquid, clear content surrounded by a slightly fibrous wall. Inside the cyst, a foreign body was evacuated and revealed to be a 5 × 2 cm piece of latex rubber excised from surgical gloves and supposedly folded to fit the underlying bony defect created by hump removal during the primary rhinoplasty procedure. Foreign material was surrounded by foreign body-type, multinucleated giant cells, and infiltration of chronic inflammatory cells inducing granulomatous reactions (Figure 1(d)).

The depressed radix residual due to the underling small bony defect was corrected by a bone autograft taken from the vomer bone exposed during concomitant closed approach septoplasty for correction of the residual nasal obstruction. The redundant skin of the radix was trimmed and accurately sutured.

The patient's postoperative course was uneventful and no external deformity remained. Nasal airflow was not affected by the procedure and the patient was satisfied with the aesthetic result.

## 3. Case 2

A 44-year-old Caucasian female with no significant medical history, who had previously undergone rhinoplasty 14 years ago, reported of a functionally satisfactory postoperative period without complications. However, three years ago, she discovered a gradually increasing 3 × 3 mm mass at the nasal radix. She also complained of persistent obstruction of nasal airflow.

**Figure 2:**
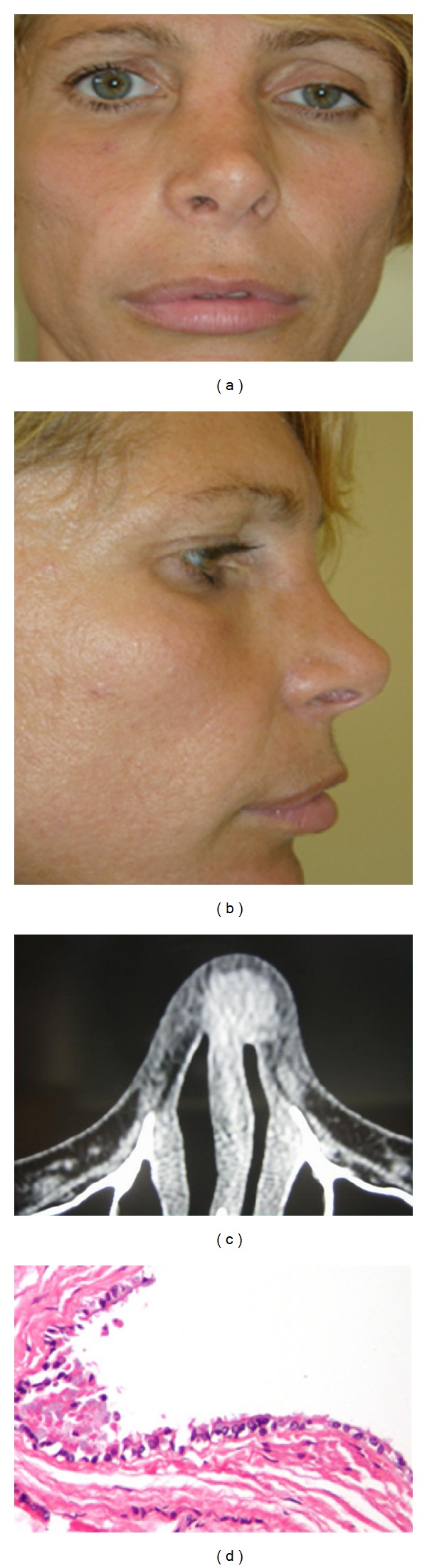
(a) Frontal view (Patient #2), (b) lateral view (Patient #2), (c) preoperative CT scan (Patient #2), and (d) pathology specimen (Patient #2).

Physical examination revealed a spherical nasal mass (1.0 × 1.0 cm), which was on palpation that was found to be firm-elastic, mobile, painless, and covered by normal skin, located on the right nasal radix (Figure 2(a)). A CT scan was performed to assess bone involvement and revealed a heterogeneous cystic mass connected to nasal bone (Figure 2(b)); the mass appeared with a poorly defined soft tissue density (90 HU) along the superior nasal dorsum. We first suspected that the cyst developed from a splinter bone of the precedent osteotomy due to the initial bone-like consistency on palpation (as reported by the patient).

The patient was admitted for removal of the mass and reconstruction of the nasal dorsum.

An open excision of the lesion was performed. The mass was found to be tightly attached to the bony dorsum and it was thus dissected down to the nasal bone and removed in en-bloc fashion. Within the intact capsule, a mucous-lined cavity filled with a thick yellow liquid was found. Histopathologic investigations revealed a benign epithelial mucous cyst containing a fragment of normal cartilage and bone (Figure 2(c)).

The lesion was diagnosed as a sequestration of a mucosal-lined nasal bone not removed at the time of hump removal.

The resulting deformity of the nasal root was corrected by using autologous bony graft from the vomer. The excessive skin was removed and sutured carefully. The patient's postoperative course was uneventful and no external deformity remained with a satisfactory final result.

## 4. Discussion

According to the literature, the different circumstances of the cases presented here suggest different pathways for cysts formation. The most accepted theories about the etiology of mucous cysts after aesthetic rhinoplasty include the presence of ectopic free mucosal graft implantation during surgical treatment, herniation of mucosa through intranasal incision, improper clearing of mucous epithelial remnants, or bony and cartilage parts during the operation [8, 9]. Other possible causes could be intrasurgical trauma and occlusion of sebaceous glands from scar tissue formation [10–13].

Inflammatory reaction to a foreign body has also previously been described as a possible cause [5, 14, 15]. In our case, considering the gross and histological findings, this reaction looks like a foreign body reaction to rubber latex material, most possibly from surgical gloves inserted 26 years ago during primary surgery. This mechanism seems akin to what has been reported for silicone implants at the nasal dorsum: silicone implants have been used for rhinoplasty since 1950 and still remains widely used implant materials in selected instances such as Asian nose augmentation rhinoplasty [16–18].

The only valid treatment of postrhinoplasty nasal dorsal cyst is the complete resection and reconstruction. We performed a direct open approach in both cases, using a glabellar horizontal incision over the cyst. We chose the direct transcutaneous route to the lesion in order to assure the adequate exposure of the upper part of the lesion. A conventional open approach to the nasal septum was unnecessary due to the absence of concomitant dorsal or tip further deformities.

Moreover, the horizontal glabellar incision seemed well tolerated, without noticeable scar formations. A closed approach using hemitransfix incision was felt sufficient to expose the septum and to avoid any dissection and possible connection between the nasal vestibule and the dorsal area. Augmentation of the residual depressed dorsum and radix with autogenous septal bone was possible.

When treating nasal dorsal masses, one first has to exclude various nasal lesions not related to surgery: neoplastic lesions, benign processes, encephaloceles, gliomas, dermoids, osteomas, lipomas, granulomatous diseases (Wegener granulomatosis, sarcoidosis, and rhinoscleroma), and infections (fungus, syphilis, and tuberculosis) [19].

In conclusion, according to the current literature, we would point out that postrhinoplasty nasal cysts do occur [10, 12, 19–21]: prevention of their formation calls for meticulous dissection before osteotomies and hump removal. It is advisable to carefully eliminate all bony, cartilagenous, epithelial or mucous tissues, and debris that may act as a foreign body at the nasal dorsum [19]. Proper closure of intranasal incisions, conservation of mucosal integrity, strict mucosal dissection, and separation in hump removal complete removal of adherent fragments of epithelium from cartilaginous grafts or flaps whenever used and pressure-free insertion of nasal dressings may further prevent cysts development.
